# Acromegaly facial changes analysis using last generation artificial intelligence methodology: the AcroFace system

**DOI:** 10.1007/s11102-025-01515-2

**Published:** 2025-04-21

**Authors:** Hatem A. Rashwan, Montserrat Marqués-Pamies, Sabina Ruiz, Joan Gil, Diego Asensio-Wandosell, María-Antonia Martínez-Momblán, Federico Vázquez, Isabel Salinas, Raquel Ciriza, Mireia Jordà, Philippe Chanson, Elena Valassi, Mohamed Abdelnasser, Domènec Puig, Manel Puig-Domingo

**Affiliations:** 1 Department of Medicine, Universitat Autònoma de Barcelona, Bellaterra, Spain; 2 Department of Computer Engineering and Mathematics, University of Rovira i Virgili, Tarragona, Spain; 3 Endocrinology Unit, Hospital de Granollers, Granollers, Spain; 4 Germans Trias Research Institute and Hospital, Service of Endocrinology and Nutrition, Badalona, Spain; 5 CIBERER group 747, Instituto de salud Carlos III, Madrid, Spain; 6 Fundamental and Medical-Surgical Nursing Department, Medicine and Health Sciences Faculty, Nursing School, Universitat de Barcelona (UB), L’Hospitalet de Llobregat, Barcelona, Spain; 7 Department of Endocrinology and Nutrition, Germans Trias i Pujol University Hospital, Badalona, Spain; 8 Spanish association of people with Acromegaly, Huesca, Spain; 9 Université Paris-Saclay, Inserm, Physiologie et Physiopathologie Endocriniennes, Assistance Publique-Hôpitaux de Paris, Hôpital Bicêtre, Service d’Endocrinologie et des Maladies de la Reproduction, Centre de Référence des Maladies Rares de l’Hypophyse, 94275, Le Kremlin-Bicêtre, France; 10 Department of Medicine, Universitat Internacional de Catalunya, Sant Cugat, Spain

**Keywords:** Acromegaly, Facial changes, Facial analysis, Artificial intelligence, Acromegaly detection

## Abstract

**Purpose:**

To describe the development of the AcroFace system, an AI-based system for early detection of acromegaly, based on facial photographs analysis.

**Methods:**

Two types of features were explored: (1) the visual/texture of a set of 2D facial images, and (2) geometric information obtained from a reconstructed 3D model from a single image. We optimized acromegaly detection by integrating SVM for geometric features and CNNs for visual features, each chosen for their strength in processing distinct data types effectively. This combination enhances overall accuracy by leveraging SVM’s capability to manage structured, quantitative data and CNNs’ proficiency in interpreting complex image textures, thus providing a comprehensive analysis of both geometric alignment and textural anomalies. ResNet-50, VGG-16, MobileNet, Inception V3, DensNet121 and Xception models were trained with an expert endocrinologist-based score as a ground truth.

**Results:**

ResNet-50 model as a feature extractor and Support Vector Regression (SVR) with a linear kernel showed the best performance (accuracy δ1 of 75% and δ3 of 89%), followed by the VGG-16 as a feature extractor and SVR with a linear kernel. Geometric features yield less accurate results than visual ones. The validation cohort showed the following performance: precision 0.90, accuracy 0.93, F1-Score 0.92, sensitivity 0.93 and specificity 0.93.

**Conclusion:**

AcroFace system shows a good performance to discriminate acromegaly and non-acromegaly facial traits that may serve for the detection of acromegaly at an early stage as a screening procedure at a population level.

## Introduction

Acromegaly is a rare, chronic disease characterized by changes in acral parts of the body, with the face being most affected by the disease [1]. These facial changes are very suggestive, and it accepted that the disease has been active for at least 10 years before diagnosis. This delay in diagnosis accounts for an important medical, psychological and social burden, impairs the quality of life of patients (QoL) and causes premature mortality [2–4]. Endocrine and metabolic illnesses [5], genetic syndromes [6], and neuromuscular diseases [7] are the most common diseases with facial manifestations. Early detection of acromegaly is crucial for prompt treatment and a better prognosis.

Artificial intelligence (AI)-driven facial recognition is advancing medical diagnostics, including acromegaly detection. Studies using facial photographs for early diagnosis show promise despite limitations [8–10]. Databases covering all disease stages enabled semi-automated detection using AI methods like support vector machine (SVM), deep learning (DL), and morphable models. Learned-Miller et al. [8] achieved 85.7% accuracy in classifying acromegaly patients with a 3D morphable model.

Here we present AcroFace, an AI-based system using DL and support vector regression (SVR) -a variant of SVM- for early acromegaly diagnosis through facial image analysis across disease stages, incorporating gender-specific features and last-generation AI techniques.

## Materials and methods

This work explores two feature types: 2D visual texture and 3D geometric patterns from facial images. A regression model estimates acromegaly risk (0–10 scale), with 0 for non-acromegaly and 10 for severe cases. The trained model obtains a score that classify images into four acromegaly levels: no disease (< 1.5), mild (1.5–5.0), moderate (5.0–8.0), and severe (≥ 8.0). No prior AI-based framework combines regression ML with visual and geometric facial features for acromegaly diagnosis.

**Fig. 1:**
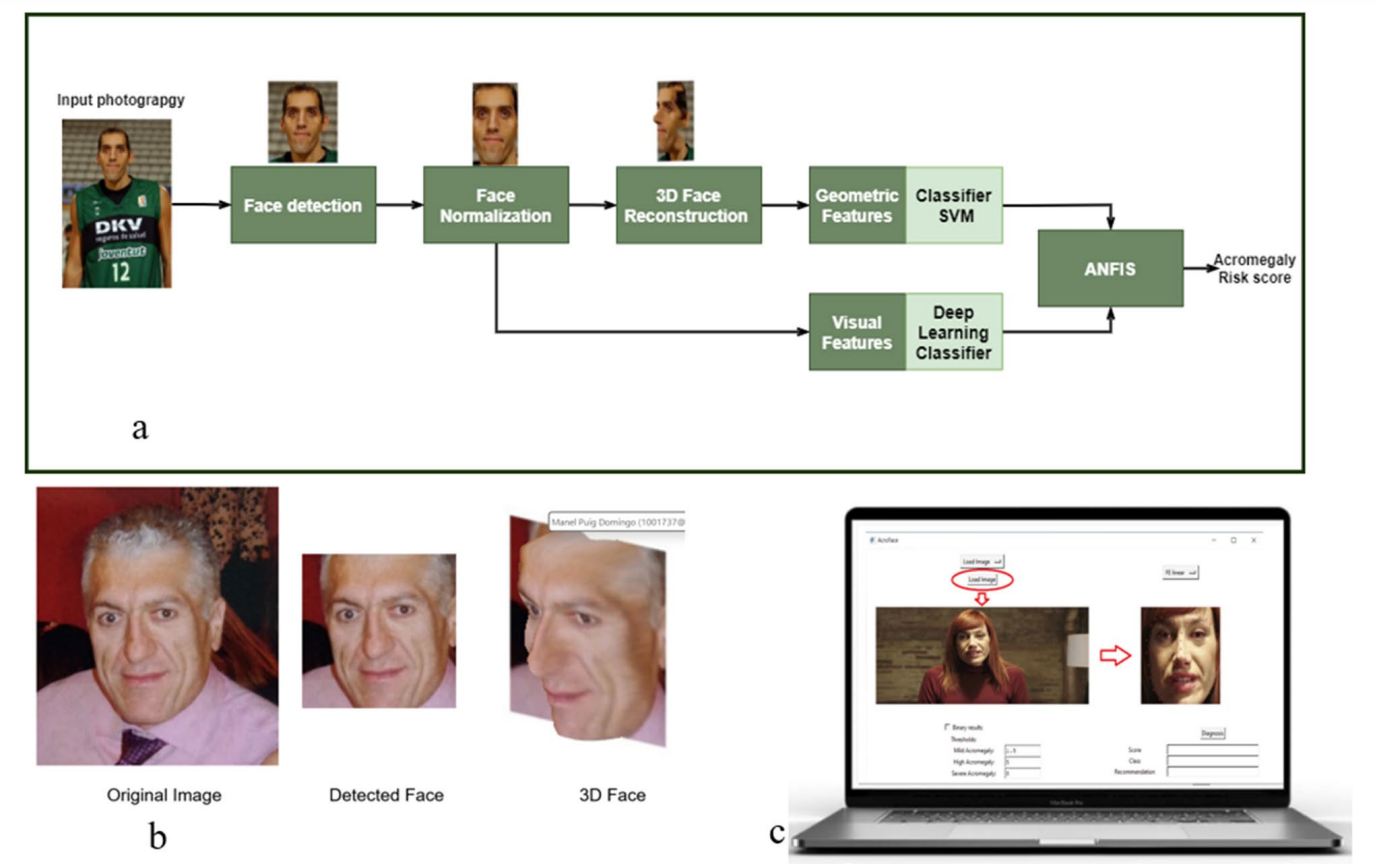
**a**: The proposed system for early detection of acromegaly. Face Detection and Normalization for loaded images. Stage1: face detection to detect faces in an input photography; stage2: face normalization to correct a rotated face to be orthogonal on the camera space; stage3: 3D face reconstruction to reconstruct a 3D face from a single image of a patient; stage 4: features extraction based on geometric from 3D faces and visual features from RGB facial images using deep learning classifier; stage5: integration of features for predicting acromegaly using a ANFIS model to provide the final score of facial images testing. **b**: Face detection and 3D reconstruction from a patient with acromegaly. **c**: Windows 10 application offering an easy-to-use interface. Users can select input options, such as “Load Image,” to upload.jpg,.jpeg, or.png files, and obtain the acromegaly score results from the analyzed face in few seconds

Figure 1a outlines the proposed acromegaly diagnosis framework. A deep learning (DL) model detects faces from input images, applying normalization to reduce appearance variance [11]. The normalization method involves aligning facial landmarks such as the eyes and mouth to standard positions, adjusting for variations in image scale, rotation, and lighting conditions, which are essential for consistent facial feature extraction. To capture global facial properties, a 3D face reconstruction was performed via volumetric convolutional neural network (CNN) regression [12] (Fig. 1b). Deep CNNs then extracted acromegaly-specific visual features and geometric biomarkers for classification.

### Dataset description

Facial images from 118 Caucasian acromegaly patients (66% women, mean age 42.8) were collected from Hospital Germans Trias, Hôpital Bicêtre, and the Spanish Acromegaly Association. Ethics approval (PI19-247) and written consent were obtained. The dataset includes 507 images from 118 patients, 86 healthy individuals (publicly sourced), and 56 with normal IGF1 levels for diversity.

The dataset included: (1) a training set with 84 subjects with acromegaly scores (0–10) assigned by 9 endocrinologists, plus 86 healthy individuals; (2) a validation set with 14 subjects for model fine-tuning and (3) a testing set with 76 subjects (56 with IGF1 measurements and 20 acromegaly patients) for performance evaluation.

Most images were captured with a mobile phone camera from printed photographs and obtained from ~ 10 years before diagnosis and yearly within 9 years pre-diagnosis; post-treatment pictures were obtained when patients were hormonally controlled or cured. The dataset includes color, digital, and scanned images, with pre-processing ensuring quality for neural network training. Images varied in angles, lighting, and background, affecting face detection. Facial scores, assigned by nine endocrinologists, showed an intraclass correlation of ~ 88% across the timeframe of pictures acquisition. Scores obtained at three different time points (10 years before diagnosis, diagnosis year and after disease stabilization or cure) were 4.3 ± 1.5, 5.8 ± 1.7, and 6.4 ± 1.5, increasing by ~ 2 points from pre-diagnosis to post-diagnosis (*p* = 0.04), with greater changes in men (6.9 ± 1.4) than women (5.3 ± 1.4), reaching significance at post-diagnosis (*p* = 0.034). Average and median scores were used for model training.

## Results

### Face detection

The face detection method FaceNet achieved an accuracy of 94%. Besides, images of low quality were excluded, resulting in a final subset of facial valid photos for subsequent analyses. In this valid facial set, the faces were clear enough to perform normalization and allowing FaceNet to achieve accuracy of 100%.

### Acromegaly grading prediction

Regression control charts face challenges with variable count, linearity, and fitting techniques, which traditional methods struggle to resolve. To improve accuracy, SVR was used with different kernels: (a) Linear, (b) Polynomial (P2–P4), (c) Gaussian RBF, and (d) Sigmoid. For visual feature extraction, deep learning models—MobileNet, InceptionV3, Xception, DenseNet121, VGG16, and ResNet50—analyzed facial textures and patterns through convolutional, pooling, and fully connected layers. Geometric features were extracted using Drira et al. model [13], detecting key landmarks (jaw, nose, eyes). CNNs then computed Euclidean distances between landmarks to create a geometric profile. These extracted features were processed by SVR with various kernels to estimate acromegaly scores (Fig. 1a).

### Evaluation metrics

To evaluate the acromegaly detection regression model, the Root Mean Square Error (RMSE), Mean Absolute Error (MAE) and R-squared (R^2^) evaluation metrics were used. The optimal value of RMSE and MAE is 0, while the optimal value of R^2^ is 1.

$$\:RMSE=\:\sqrt{\frac{1}{n}{\sum\:}_{j=1}^{n}{\left({y}_{j}-{\widehat{y}}_{j}\right)}^{2}}\:,\:\:\:\:$$

$$\:MAE=\:\frac{1}{n}{\sum\:}_{j=1}^{n}\left|{y}_{j}-{\widehat{y}}_{j}\right|,\:\:\:\:\:$$

$$\:\:{R}^{2}=\frac{{\sum\:}_{j}^{}{\left({y}_{j}-{\widehat{y}}_{j}\right)}^{2}}{{\sum\:}_{j}^{}{\left({y}_{j}-\underset{\_}{y}\right)}^{2}}$$

where *n = number of samples*,* y_j = Ground Truth score of sample j*,

*y ^_j = Predicted score for sample j*,* and _y = Average of Ground Truth scores.*

We also calculated the classification rate of the proposed system. The classification accuracy is computed based on an error threshold, so if the difference between the ground truth and the predicted score is less than or equals to a specific threshold, we considered it as a success; otherwise, it is a failure.

$$\:System\:Accuracy=\frac{1}{n}{\sum\:}_{j=1}^{n}\left(\left|{y}_{j}-{\widehat{y}}_{j}\right|<\delta\:\:\right)$$

where the value of |y j-y ^_j| <δ equals 1 when the condition is met and 0 when it is false. The thresholds used for this test are (a) Threshold 1 (δ^1^): (1.25)^1^ = 1.25, (b) Threshold 2 (δ^2^): (1.25)^2^ = 1.5625 and (c) Threshold 3 (δ^3^): (1.25)^3^ = 1.9531. The threshold values are progressively increasing powers of 1.25, representing increasing levels of tolerance in our accuracy assessment. This stepwise increase allows us to demonstrate the robustness of our algorithm under varying strictness levels of match criteria between predicted and actual values.

### Performance comparison of the visual and geometric features to the average and median of 9 endocrinologists

The detection of acromegaly using visual and geometric features was compared. The system’s predicted scores were evaluated using RMSE, MAE, and R², with the average and median scores from the 9 endocrinologists as the ground truth. Geometric features resulted in higher RMSE and MAE errors compared to visual features, achieving lower errors of 1.6915 for RMSE and 1.2642 for MAE. The R² metric was also poor for geometric features, indicating its inadequacy for detecting acromegaly risk. In contrast, visual features showed an R² close to 1, suggesting they are effective for detecting acromegaly. ResNet-50 outperformed other backbone networks. Visual features yielded low accuracy (best: 58% with sigmoid kernel), while SVR with a linear kernel achieved the highest accuracy (δ1: 70%, δ3: 84%). Increasing the threshold between actual and predicted scores could further improve accuracy.

### Performance comparison of visual and geometric features to an expert endocrinologist

We compared our model to the expert endocrinologists scores, using them as ground truth for training deep models (ResNet-50, VGG-16, MobileNet, Inception V3, DenseNet121, Xception), testing on 10 healthy cases and 10 patients. An improvement of 25% in RMSE, MAE, and R² was found compared to models trained on average/median scores from 9 endocrinologists. ResNet-50 with SVR (linear kernel) performed best, followed by VGG-16. Geometric features underperformed, with SVR (RBF kernel) yielding the best results for them.

Visual features outperformed geometric features in accuracy: sigmoid SVR with geometric features achieved δ1, δ2, δ3 accuracies of 64%, 74%, and 78% respectively, thus achieving a 6% improvement, while linear SVR with visual features achieved δ1: 75% and δ3: 89%, thus implying a 5% improvement. When validation with a second cohort was performed (32 patients, 44 controls), the system achieved precision: 0.90, accuracy: 0.93, F1-Score: 0.92, sensitivity: 0.93, and specificity: 0.93.

### Performance with a control set

To assess AcroFace’s ability to identify non-acromegaly cases, we tested it on a control dataset of 56 facial images from healthy individuals with normal IGF1 levels. The dataset included diverse ages, genders and facial features to ensure robust specificity evaluation. The test was conducted blindly, with consistent preprocessing (e.g., facial alignment, normalization) and no manual intervention. With a threshold of 1.5, AcroFace achieved 92.8% specificity and a 7.14% false positive rate (FPR) as the percentage of controls misclassified as acromegaly positive. Increasing the threshold to 2.0 improved specificity to 100%.

### Graphical user interface

A Windows 10 application was developed to integrate all system components, offering an easy-to-use interface. Users can select input options, such as “Load Image,” to upload.jpg,.jpeg, or.png files. The system automatically detects and normalizes the face, displaying it with landmarks on the right side (Fig. 1c). After loading and detecting the face, users can start the analysis, choosing between SVR kernels or using the default, most accurate model. The application provides two classification options: a 4-category of acromegaly risk classification (No-acromegaly, mild, high, very high facial phenotype risk score) or a binary classification (acromegaly risk or not).

## Discussion

We have advanced the field with AcroFace, a CNN-based system that not only achieves approximately 90% accuracy but also introduces a novel integration of SVM and CNN models for enhanced facial analysis. This dual-mode approach allows for precise detection of acromegaly, effectively handling both geometric and visual features, thereby significantly outperforming the 85.7% accuracy achieved by Learned-Miller et al. [8] with a 3D morphable model, Jackson et al [13] 3D face reconstruction model which achieved 86%, outperforming physicians (26%) and the 81% by a Gabor wavelet-based method [14] as well as Gencturk et al [15] which used combined local binary patterns (LBP) and Manhattan classifiers (97% accuracy). Despite utilizing a more focused dataset of 118 cases, our system demonstrates comparable efficacy to larger studies such as Kong et al. [16] with 527 cases and 596 controls, and Wei et al. [17] with 896 patients and 11,447 controls, achieving 96% and 94.8% accuracy, respectively. AcroFace stands out not only for its high accuracy but also for its user-friendliness and adaptability in clinical settings, making it an indispensable tool for the early and accurate diagnosis of acromegaly, which is critical for improving patient outcomes.

AI-based acromegaly screening remains underutilized despite recent advances [18–27]. Traditional 2D facial image analysis has proven effective, and new AI techniques allow screening from standard photos without strict imaging conditions. This enhances screening feasibility at a population level, which, to the best of our knowledge, still has not been performed. These systems may also aid in tracking post-treatment disease evolution/regression. Notably, our dataset showed that facial changes were identifiable up to 10 years before diagnosis, indicating that the disease may be running even for a longer period of time than previously assumed.

AcroFace requires no human intervention—only a frontal face image—making it adaptable even for self-screening apps, which may eventually broaden the screening possibilities. However, limitations include a relatively small dataset and an ethnic bias toward Caucasian subjects. While AcroFace showed high accuracy, occasional misclassifications highlight the need for further validation across diverse populations.

Training medical professionals is time- and cost-intensive, while AI can rapidly develop expertise. AcroFace fully automates screening, providing an efficient, scalable solution. Given acromegaly’s slow progression and frequent late diagnoses, AI-based tools like AcroFace could transform early detection, reducing complications and improving patient outcomes.

## Data Availability

No datasets were generated or analysed during the current study.
